# Whole *Pichia pastoris* Yeast Expressing Measles Virus Nucleoprotein as a Production and Delivery System to Multimerize *Plasmodium* Antigens

**DOI:** 10.1371/journal.pone.0086658

**Published:** 2014-01-27

**Authors:** Daria Jacob, Claude Ruffie, Myriam Dubois, Chantal Combredet, Rogerio Amino, Pauline Formaglio, Olivier Gorgette, Gérard Pehau-Arnaudet, Charline Guery, Odile Puijalon, Jean-Christophe Barale, Robert Ménard, Frédéric Tangy, Monica Sala

**Affiliations:** 1 Institut Pasteur, Viral Genomics and Vaccination Unit, Paris, France; 2 CNRS, URA3015, Paris, France; 3 Institut Pasteur, Malaria Biology and Genetics Unit, Paris, France; 4 Institut Pasteur, Molecular Immunology of Parasites Unit, Paris, France; 5 CNRS, URA2581, Paris, France; 6 Institut Pasteur, PFMU, Paris, France; 7 CNRS, UMR3528, Paris, France; 8 Institut Pasteur, Malaria Biology and Genetics Unit, Team Malaria Targets and Drug Development, Paris, France; Instituto Butantan, Brazil

## Abstract

Yeasts are largely used as bioreactors for vaccine production. Usually, antigens are produced in yeast then purified and mixed with adjuvants before immunization. However, the purification costs and the safety concerns recently raised by the use of new adjuvants argue for alternative strategies. To this end, the use of whole yeast as both production and delivery system appears attractive. Here, we evaluated *Pichia pastoris* yeast as an alternative vaccine production and delivery system for the circumsporozoite protein (CS) of *Plasmodium*, the etiologic agent of malaria. The CS protein from *Plasmodium berghei* (*Pb*) was selected given the availability of the stringent C57Bl/6 mouse model of infection by *Pb* sporozoites, allowing the evaluation of vaccine efficacy *in vivo*. PbCS was multimerized by fusion to the measles virus (MV) nucleoprotein (N) known to auto-assemble in yeast in large-size ribonucleoprotein rods (RNPs). Expressed in *P. pastoris*, the N-PbCS protein generated highly multimeric and heterogenic RNPs bearing PbCS on their surface. Electron microscopy and immunofluorescence analyses revealed the shape of these RNPs and their localization in peripheral cytoplasmic inclusions. Subcutaneous immunization of C57Bl/6 mice with heat-inactivated whole *P. pastoris* expressing N-PbCS RNPs provided significant reduction of parasitemia after intradermal challenge with a high dose of parasites. Thus, in the absence of accessory adjuvants, a very low amount of PbCS expressed in whole yeast significantly decreased clinical damages associated with *Pb* infection in a highly stringent challenge model, providing a proof of concept of the intrinsic adjuvancy of this vaccine strategy. In addition to PbCS multimerization, the N protein contributed by itself to parasitemia delay and long-term mice survival. In the future, mixtures of whole recombinant yeasts expressing relevant *Plasmodium* antigens would provide a multivalent formulation applicable for antigen combination screening and possibly for large-scale production, distribution and delivery of a malaria vaccine in developing countries.

## Introduction

Antigen delivery is a major issue in the success of vaccines. Although only a few adjuvants are licensed [1], a large array of chemical-based new adjuvants or immunostimulants for vaccine antigens are currently developed [2]. However, several concerns about the safety of using chemicals in association with vaccines are raised [3], [4], [5], [6], [7]. Therefore, alternative delivery strategies need to be developed. Among them, the use of attenuated [8] or inactivated [9], [10], [11] yeast is emerging. Yeast-based vaccines elicit both humoral and cell-mediated immune responses in the absence of adjuvants [9], [10], [11], [12]. Heat-killed yeasts have been shown to protect mice against systemic aspergillosis and coccidioidomycosis [13], or to provide sterile protection to chicken towards infectious bursal disease [14]. Recombinant yeasts are currently developed as vaccine candidates against HBV and HCV in humans [15] or leukemia [16]. Whole yeasts activate dendritic cells (DCs) and are efficiently taken up through fungipods or phagocytic synapses on DCs [17], [18]. Both mannose and Dectin-1 receptors mediate the interaction between human DCs and the most biotechnologically relevant yeasts: *Saccharomyces cerevisiae* (*S. cerevisiae*) and *Pichia pastoris* (*P.pastoris*) [19]. DCs can distinguish direct fungal contacts from soluble fungal-derived components through the Dectin-1 pattern-recognition receptor [18]. Thus, activated DCs become potent presenting-cells for antigens expressed in recombinant yeast, and efficiently deliver antigens into both MHC class I and class II pathways. Hence, yeast-DC interplay provides a strong adjuvant effect on antigen immunogenicity [9], [10], [11], [12].

Multimerization of monomeric antigens was also largely demonstrated to amplify their immunogenicity through increased uptake by DCs [20], [21], [22], [23]. Thus, combining the antigen multimerization with expression in whole yeast would be even more advantageous. Several multimeric proteins, generally from viral origin, have been used as delivery systems [24], [25], [26], [27]. The nucleoprotein (N) of measles virus (MV), which composes the viral helical nucleocapsid [28], [29], has the capacity to auto-assemble around any RNA molecule in the cytoplasm of cells in which this protein is expressed (mammalian [30], bacterial [31] or yeast [32]) with a ratio of 1 N molecule to 6 ribonucleotides. This gives rise to helical, highly stable, and multimeric ribonucleoprotein rods (RNPs) similar in shape and diameter to RNPs present in MV viral particles [33]. The expression of MV-N protein in *P. pastoris* GS115 yeast strain induces the formation of high amounts of these RNPs visible in the cytoplasm by electron microscopy [32]. Thus, whole recombinant yeast expressing MV-N appears as a promising delivery system for multimerizing vaccine antigens.

Currently tested whole yeast-based vaccine candidates are based on *S. cerevisiae* yeast. In 2009 the entire genome of *P. pastoris* yeast (GS115 strain) was sequenced [34]. This encouraged the development of *P. pastoris* as bioreactor in vaccinology. Indeed, *P. pastoris* offers many advantages compared to *S. cerevisiae*, such as the stringent control of protein production through a strong inducible promoter and the reduced length of the oligosaccharide chains eventually added post-translationally to transgenic proteins. Moreover, terminal *alpha-*1,3 glycan linkages on glycosylated proteins, which are responsible for hyper-antigenicity effects of antigens produced in *S. cerevisiae* [35], are not formed in *P. pastoris*.

To evaluate a new vaccine platform based on recombinant whole yeast as antigen delivery vector for multimerized antigens, we used a malaria animal model. Despite major research efforts, an efficient malaria vaccine is still not available [36]. Hence, it is worthwhile to investigate new approaches. Malaria is caused by the multiplication of *Plasmodium* parasites in the blood after injection in the skin by a mosquito. The parasite form in the skin, called sporozoite, invades hepatocytes to develop into red blood cell (RBC) infecting forms, during the pre-erythrocytic phase of infection [37]. The *Plasmodium* sporozoite is covered with the monomeric circumsporozoite protein (CS), the leading vaccine candidate against the pre-erythrocytic stage of *Plasmodium* [38], [39]. Antibodies to CS [40], [41], [42], as well as specific CD8^+^ T cells [42], [43], [44], are known to protect against sporozoite challenge in animal models, primarily rodents. In humans, RTS,S - the most advanced malaria vaccine candidate based on the CS antigen - provides 30–56% protection [45]. In RTS,S, CS multimerization is achieved by its association to hepatitis B virus-like particles.

Here, CS multimerization is obtained by fusing the CS from *Plasmodium berghei* (*Pb*) ANKA strain (a rodent-infective species) to the N protein from MV. We generated recombinant *P. pastoris* yeast expressing N or PbCS alone, or N-PbCS RNPs, and characterized the size, shape and cellular localization of these RNP structures. Heat-inactivated recombinant yeasts were used to immunize C57Bl/6 mice, the most susceptible laboratory mice to *Pb* sporozoite infection [46]. In this model, most animals die during the first 10 days post infection presenting symptoms of experimental cerebral malaria, or later as a consequence of extreme red blood cell infection [47]. Following subcutaneous immunization with whole yeast expressing N-PbCS RNPs then intradermal challenge with *Pb* sporozoites, the onset of blood infection was significantly delayed, and animal survival was prolonged. The profile of anti-PbCS IgG antibodies reflected unbiased contributions of both Th1 and Th2 immune responses.

## Results

### Expression of Measles virus Nucleoprotein in *Pichia pastoris*

**Figure 1:**
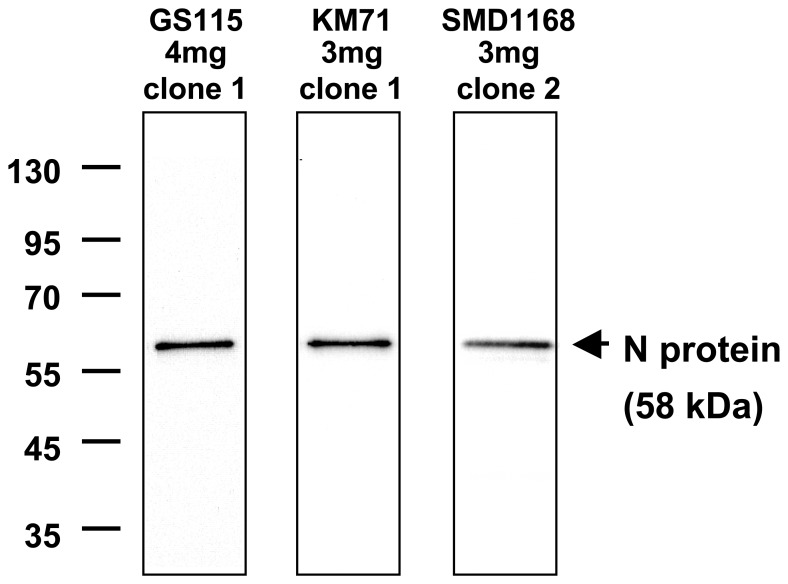
Expression of N protein in GS115, KM71 and SMD1168 *P. pastoris* strains. Concentrations of Geneticin in selection plates for the specific clones, and clone numbers, are indicated. Yeast lysates were diluted 1/600 before loading on western blot.

The nucleotide sequence encoding the nucleoprotein (N) of measles virus vaccine (MV) Schwarz strain [48] was optimized for expression in *P. pastoris* and cloned into the pPIC3.5K plasmid under the control of the methanol-inducible AOX1 promoter. Three strains of *P. pastoris* (the commonly used GS115 and KM71, as well as SMD1168, which is deficient in proteinase A activity) were transformed with the recombinant plasmid and 10 positive clones per strain were amplified. A first kinetic study of N expression was performed by western blot analysis of yeast lysates. The optimal time point for N expression was found to be 54 hours (h) after methanol induction for the three *P. pastoris* strains. The best N-expressing clone for each strain was then selected by western blot analysis of yeast lysates collected 54 h after induction. These clones showed the highest expression of full-length undegraded N of comparable weights (Figure 1). The N expression was further characterized in the GS115, KM71, and SMD1168 best clones by Bradford analysis and quantitative western blot. The N protein was expressed at the predicted apparent molecular weight with no visible degradation or processing. The amount of N protein expressed was around 1 µg per yeast unit (YU). In the GS115 and KM71 strains, N production accounted for as much as 24% of total soluble proteins (TSP), while this was only 14% in the SMD1168 strain (Table 1). The expression levels in GS115 and KM71 were in the same range as previously shown for these strains [32].

**Table 1:** Amount of N protein expressed in *P. pastoris* GS115, KM71 and SMD1168 strains. One YU corresponds to 10^7^ yeast cells.

	*P. pastoris* strain		
	GS115	KM71	SMD1168
**N protein amount per YU (µg)**	1.25	1.14	0.87
**Total soluble protein (TSP)** **per YU (µg)**	5.11	4.63	5.98
**N protein/TSP (%)**	24	24	14

### Expression of N-PbCS in SMD1168 *Pichia pastoris*

**Figure 2:**
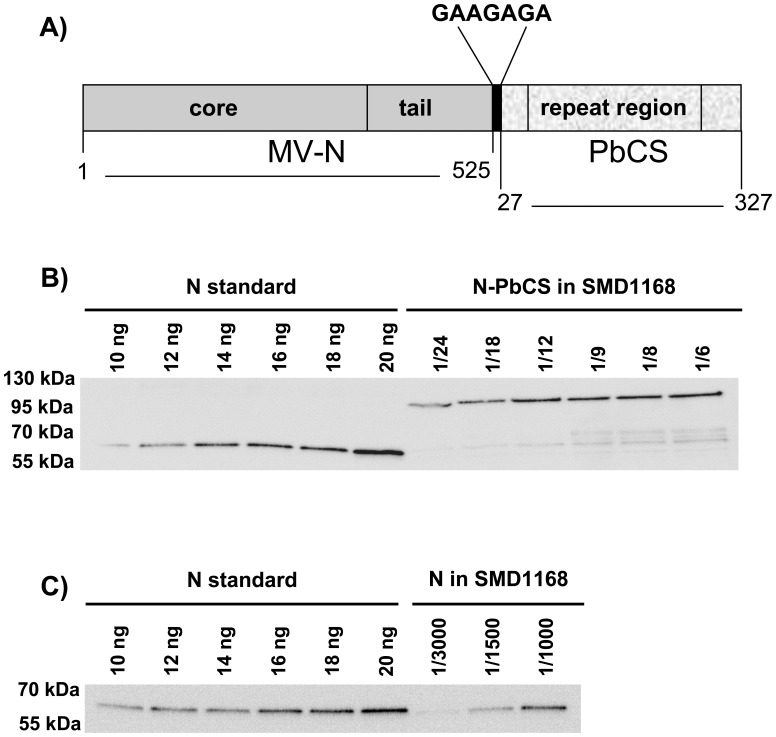
Expression of N-PbCS in *P. pastoris*. (A) Schematic representation of N-PbCS fusion protein. MV-N (dark grey) is composed of a core domain in N-terminal and a unstructured tail domain in C-terminal [69]. The GAAGAGA linker is in black. PbCS (light grey) corresponds the central repeat region flanked by major portions of the N-terminal and C-terminal domains of the protein [39]. Amino acids numbering are given according to N from the MV Schwarz vaccine strain and PbCS from the *Pb* ANKA strain. For sequence details, see Figure S1. (B) Quantitative western blot analysis of SMD1168 expressing N-PbCS or (C) N. In (B) and (C), yeast lysates were diluted as indicated, the MV-N protein was used as a standard with increasing concentrations and western blots were probed with an anti-N antibody.

To test the possibility of using MV-N-based RNPs as carrier to multimerize heterologous antigens in malaria vaccine prototypes, we fused the circumsporozoite (CS) antigen [49] from *Pb* (ANKA strain) to the C-terminus of MV-N through a linker of 7 amino acids (Figure 2A; Figure S1; [50]). The choice of using *Pb* opens access to the mouse animal model to evaluate the immunogenicity and efficacy of the vaccine prototype by vaccination and parasite challenge [46], [47]. GS115, KM71 and SMD1168 *P. pastoris* strains were transformed by pPIC3.5K bearing the N-PbCS-encoding gene under the control of the AOX1 methanol-inducible promoter. Induction of N-PbCS in GS115 and KM71 strains resulted in the rapid degradation of the fusion protein (data not shown), while the full-length fusion protein was correctly produced in SMD1168 strain (Figure 2B). The maximum expression of N-PbCS was obtained at 54 h after induction. In the best N-PbCS expressing clone selected by qualitative and quantitative western blot, the expression level of fusion protein (12 ng/YU) was 73 times lower than N alone (871 ng/YU) in the same strain (Table 1; Figure 2C). Thus, full length N-PbCS fusion protein could only be expressed in SMD1168 *P. pastoris* and at a nearly 2-log lower level than the N protein alone. In order to obtain control yeast expressing the monomeric form of the *Plasmodium* antigen, an SMD1168 clone producing PbCS alone was generated and selected as previously described. PbCS yeast showed a comparable expression level of the antigen alone (16 ng/YU) to that of the N-PbCS yeast (12 ng/YU). The replication kinetics of recombinant SMD1168 yeasts expressing N, PbCS or N-PbCS were strictly comparable in all tested culture media (YPD, BMG and BMM), and no macroscopic phenotype difference was observed between recombinant and wild type SMD1168 yeasts.

### Production of High Molecular Weight N-based Ribonucleoproteins in *Pichia pastoris*

**Figure 3:**
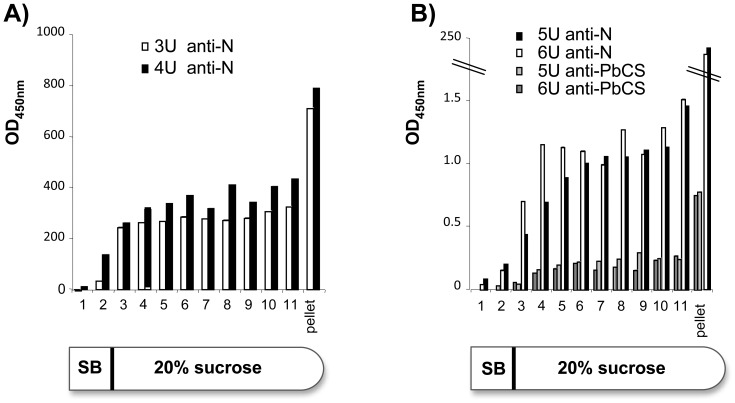
ELISA quantification of N or PbCS proteins in ultracentrifugation (U) fractions and pellets of SMD1168 lysates expressing N alone at 871 ng/YU (A) or N-PbCS at 12 ng/YU (B). Yeast cultures, lysates and ultracentrifugations were performed in duplicate (3 U and 4 U for N expressing yeast, and 5 U and 6 U for N-PbCS yeast). Values correspond to optical densities at OD_450 nm_ (taking OD_620 nm_ as reference) multiplied by sample dilutions. SB: suspension buffer.

The MV-N nucleoprotein has the capacity to auto-assemble around RNA in the cytoplasm of mammalian, bacterial or yeast cells [30], [31], [32]. To assess whether N and N-PbCS were assembled into high-molecular weight RNPs in SMD1168 *P. pastoris*, yeast lysates were ultracentrifuged on 30% sucrose and the presence of N and PbCS proteins was quantified in fractions and pellets (Figure 3). Although N multimerization may impact the affinity of RNPs of different size for anti-N antibodies used for quantification, a comparison of N amounts can be performed among fractions at similar levels in N and N-PbCS samples. For both recombinant yeasts, the profile of N and N-PbCS distribution on sucrose showed the presence of monomeric or oligomeric N in the upper fractions, multimeric forms in the middle fractions and highly multimeric RNPs in the pellets. Notably, we estimated by calculation routine (see Materials and Methods) that RNPs present in the pellets were heavier than 36,502 kDa, assembling more than 628 N molecules. The 2-log difference in read out between the two recombinant yeasts relates to the 2-log difference in N expression level between N and N-PbCS yeasts (871 ng/YU versus 12 ng/YU).

Remarkably, in N-PbCS recombinant yeast, N was predominantly found in the pellet. Quantitative western blot analysis of N-PbCS pellets demonstrated that RNPs were mainly constituted of N (70–80%) and that full length N-PbCS protein represented 10–20% of total N. In addition, recombinant RNPs contained 10% of degraded N-PbCS proteins (data not shown). The imbalanced profile of N-PbCS yeast may be due to RNP pull down by interaction of PbCS antigen with subcellular elements or alternatively to RNPs stabilization by N-PbCS fusion resulting in highly multimeric RNPs. Heterologous antigen expression did not modify yeast RNA and TSP patterns as compared to wild type yeasts (Figure S2). Theses profiles were maintained independently of yeast amounts loaded on sucrose (data not shown).

**Figure 4:**
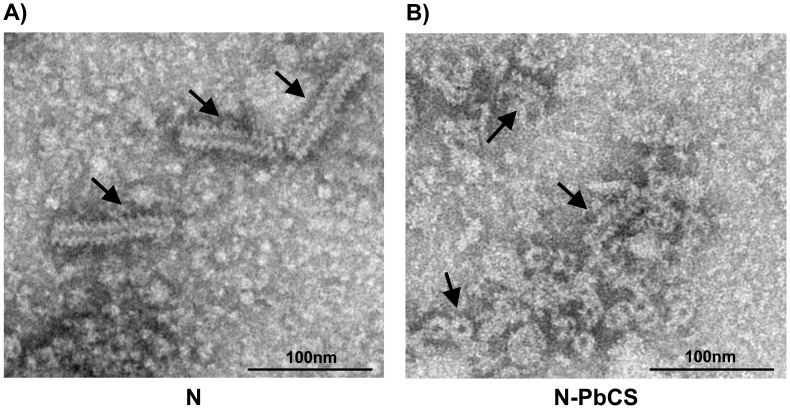
Electron microscopy analysis of yeast lysates from SMD1168 *P. pastoris* expressing N (A) or N-PbCS (B). Scale bars are indicated. Black arrows highlight RNP rod and ring structures.

To look for the structure of RNPs, yeast lysates were observed by electron microscopy (EM). In the N recombinant SMD1168 yeast, we found numerous RNPs with herringbone shape of 20–22 nm diameter and variable rod length of 30 to 200 nm (Figure 4A). This finding was similar to that previously described in recombinant *P. pastoris* GS115 strain [32] and in mammalian cells infected by MV [29]. In the N-PbCS sample, RNPs appeared less rigid than N-only RNPs with length spanning from 30 to 70 nm. Most N-PbCS RNPs were detected in microenvironments where discrete rings were visible next to rod-like structures (Figure 4B). Because of the lower expression level of N-PbCS as compared to N, the N-PbCS clarified lysate was submitted to two ultracentrifugation rounds to concentrate N-PbCS structures, while N sample was not. This explains the differences observed in average rod length in both samples, as previously observed for N RNPs in yeast [32]. Moreover, after the first round of ultracentrifugation we selected the fractions containing RNPs of around 333 N molecules, which represented the major population as compared to shorter or longer RNP structures present in the other ultracentrifugation fractions or pellet. This assumption was made taking into account that 1 N molecule associates with 6 RNA ribonucleotides [29], and that eukaryotic mRNAs have an average size of 2 kb [51]. RNP selection was then performed on the basis of a sedimentation calculation routine (see Methods). The presence of lighter and heavier N-PbCS RNPs in lysate as well as of N-PbCS fusion protein in selected RNPs was put in evidence by anti-PbCS/anti-N sandwich ELISA on all 1 ml fractions taken from the top to the bottom of tubes from both ultracentrifugation rounds (data not shown).

**Figure 5:**
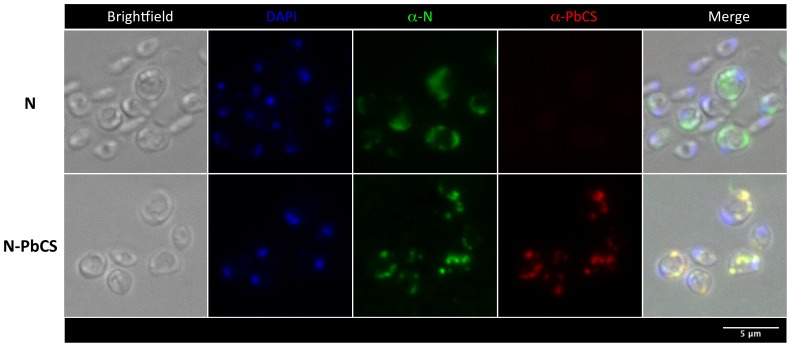
Immunofluorescence analysis of N or N-PbCS expression in yeasts (N staining in green, PbCS staining in red and nuclei in blue). Each image is the maximal intensity projection of three consecutive focal planes spaced 0.5 µm apart.

Immunofluorescence analysis of N or N-PbCS recombinant SMD1168 yeasts showed that RNPs localized in large and compact cytoplasmic inclusions, as previously observed for N alone in GS115 *P. pastoris* [32], and that N inclusions co-localized with PbCS in N-PbCS yeasts (Figure 5).

### Heat-inactivation of *Pichia pastoris*

The development of *P. pastoris* for whole recombinant yeast vaccine, as an alternative to *S. cerevisiae,* needs to set up a protocol of heat-inactivation ensuring the death of vegetative yeast cells before their use. A recombinant *S. cerevisiae* previously tested in Phase IIb trial was heat-inactivated at 56°C for 1 h [15]. As this protocol only partially inactivated *P. pastoris* GS115, we evaluated a series of inactivation temperatures and time of treatment to achieve complete impairment of yeast growth. Survival of *P. pastoris* GS115, KM71 and SMD1168 yeasts after different heat-treatments was analyzed by culture both on plates and in liquid medium for 7 days at 30°C. The total loss of reproductive capacity was associated to the lack of metabolic activities, as assessed by methylene blue viability test. Complete growth impairment for the three strains was obtained following heat-inactivation at 58–60°C for 45–60 minutes (Figure S3). For the next experiments, we used 60°C for 45 minutes.

### Evaluation of the Immunogenicity and Efficacy of Whole Recombinant N-PbCS SMD1168 Yeast Vaccine in the *Plasmodium berghei -* C57Bl/6 Mouse Model

**Figure 6:**
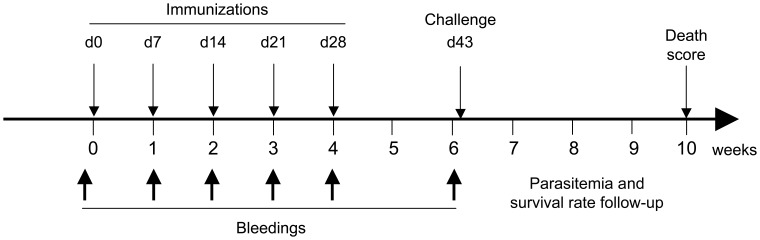
Schematic representation of the immunization protocol. Immunization and challenge schedule is given in days (d) and bleeding time points in weeks.

To evaluate the immunogenicity and efficacy of whole recombinant SMD1168 *P. pastoris* expressing MV-N-based RNPs as carrier of PbCS antigen, we used the C56Bl/6 mouse model of *Pb* infection, a highly stringent animal model for severe rodent malaria [46]. Immunizations were performed by five subcutaneous injections (once every week) of 30 YU heat-inactivated SMD1168 *P. pastoris* in the absence of accessory adjuvant (Figure 6). Two separate experiments were independently performed with five groups of mice in each (6 and 8 mice per group, respectively). The first group was inoculated with yeast expressing N-PbCS (360 ng of N-PbCS corresponding to 230 ng of N and 130 ng of PbCS antigens); the second group with yeast expressing PbCS only (130 ng of PbCS antigen); the third group with yeast expressing N only (230 ng of N); the fourth group with wild type (WT) yeast. In groups two and three, recombinant yeast was diluted with wild type SMD1168 yeast to adjust the amounts of PbCS or N with respect to the N-PbCS group. A last group was kept unvaccinated, but housed in parallel (naive). Bleedings were performed every week during immunization and just before challenge (day 42). All groups of mice were challenged at day 43 with 6,000 GFP^+^ *Pb* sporozoites, then parasitemia and mouse survival rate were monitored. GFP^+^ *Pb* sporozoites were used to facilitate parasitemia determination by flow cytometry (these modified *Pb* sporozoites have similar growth and infectivity rates than wild type parasites [52]).

**Figure 7:**
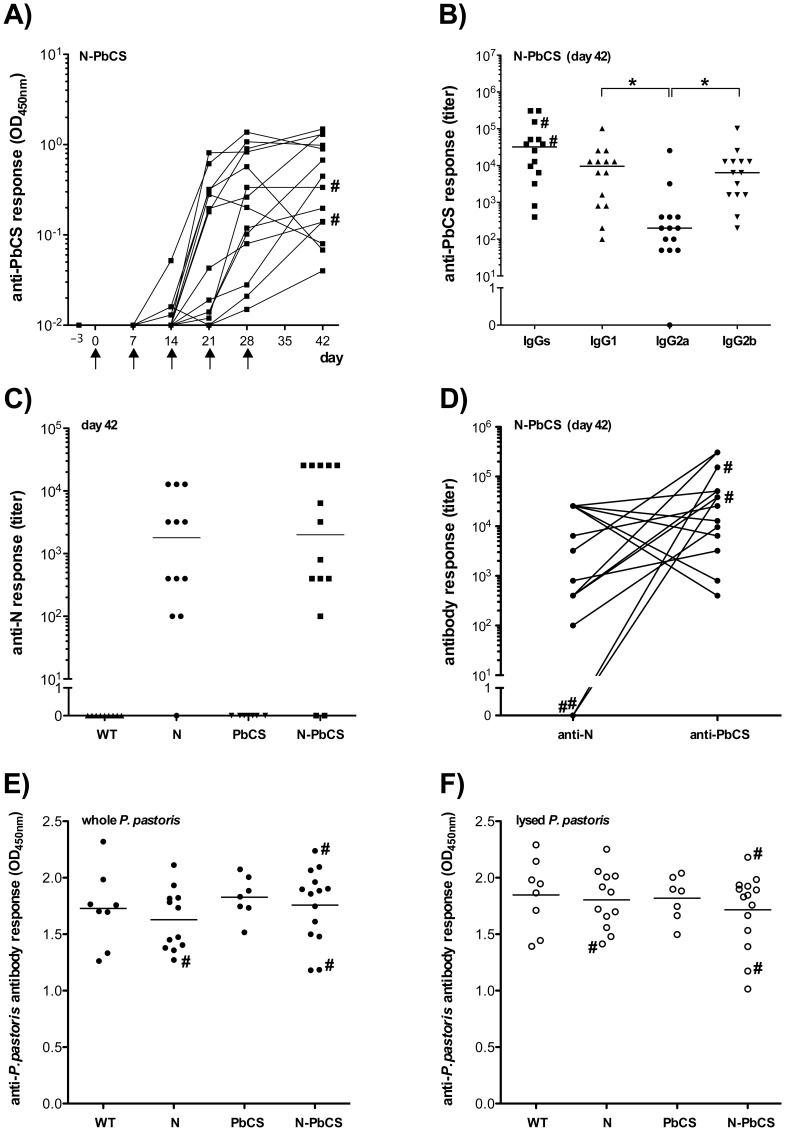
Humoral responses elicited in mice after immunization. (**A**) Kinetics of anti-PbCS IgG responses in mice immunized with N-PbCS yeasts. OD_450 nm_ are expressed in log_10_ scale. Black arrows indicate immunization schedule. (**B**) Isotyping of humoral IgG responses at day 42 in mice immunized with N-PbCS. The bars correspond to median values per group. Asterisks (*) indicate significant median differences (p<0.05; Mann-Whitney nonparametric test). (**C**) Anti-N IgG titers in mice serums collected at day 42 after immunization with WT yeast or yeasts expressing N, PbCS or N-PbCS. Median values were compared by the Wicoxon Two Sample Test (p = 0.4558). (**D**) Antibody titers of N-PbCS mice are compared (see panels B and C). The lines associate titers from the same mouse. (**E**) Anti-*P. pastoris* IgG responses towards whole yeast. (**F**) Anti-*P. pastoris* IgG responses towards lysed yeast. The bars in (E) and (F) correspond to mean values per group. Hash sign (#) indicates anti-N antibody-negative mice from the N-PbCS group (see panel C).

**Figure 8:**
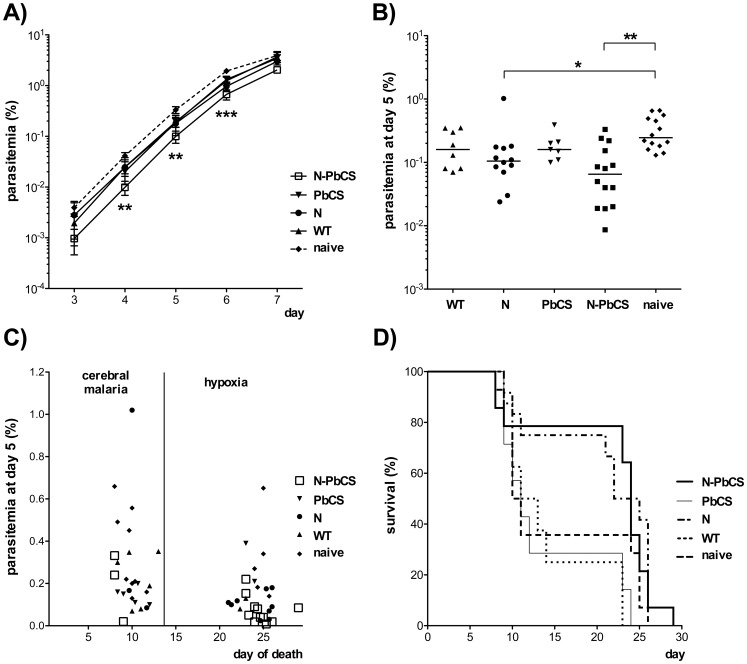
Experimental challenge of immunized mice. (**A**) Mean and standard deviations log_10_ values of parasitemia in mice immunized by N-PbCS, PbCS, N or WT yeast, and in non-immunized mice following infection with 6,000 GFP^+^ *Pb* sporozoites. Blood parasitemia is expressed in log_10_ scale as the percentage of infected red blood cells (iRBCs) out of total RBCs along the first 7 days follow up. Asterisks (*) indicate the significance level of the Mann-Whitney nonparametric test: two symbols correspond to p<0.005 and three to p<0.0005. (**B**) Parasitemia at day 5 post-challenge. Bars correspond to medians. Asterisks (*) indicate significant median differences (one symbol for p<0.05 and two for p<0.005; Mann-Whitney nonparametric test). (**C**) Inverse correlation between the day of death (x axis) and the percentage of iRBCs per total RBCs (y axis; arithmetic scale) per mouse. The cause of death is given in the upper part of the graph. (**D**) Survival curves of immunized mice after challenge with 6,000 GFP^+^ *Pb* sporozoites.

The results obtained in the two immunization experiments were comparable, showing the robustness of outputs despite two independent sporozoites and recombinant yeasts preparations. Therefore, to improve statistical robustness, we pooled the results from both experiments into a single analysis (Figure 7 and Figure 8).

All mice immunized with N-PbCS yeast raised anti-PbCS antibodies after the third immunization whose level still increased in half of the animals after the last injection (Figure 7A). Median IgG titer reached 3×10^4^ at day 42 when immunization was completed (Figure 7B). By contrast, mice immunized with yeast expressing PbCS only remained negative all over the follow up (data not shown). Retrieving anti-PbCS antibody response despite a very low antigen dose (130 ng PbCS per injection) in the N-PbCS group as compared to the PbCS group demonstrates the self-adjuvant capacity of this vaccine platform, notably the positive impact of antigen multimerization by N-based RNPs.

As IgG subclasses mediate different immune effector functions depending on their structures [53], we determined the IgG subclass responses to PbCS in N-PbCS immunized mice before challenge (Figure 7B). There was no Th1 or Th2 polarization of the humoral immune response since the IgG1 response was statistically comparable to IgG2b, and the relevant difference between IgG1 and IgG2a (p<0.05) was compensated in the Th2/Th1 bias by the IgG2b response (Mann-Whitney nonparametric test). Remarkably, the detection of significant IgG1 and IgG2a/b humoral responses underlines the elicitation of both Th1 and Th2 cytokine environments after vaccination with the whole recombinant N-PbCS yeast in the absence of accessory adjuvants.

Anti-N IgG antibodies became detectable after the third injection in mice immunized with N or N-PbCS yeasts and reached highest titers at day 42. The anti-N response at day 42 (Figure 7C) was statistically comparable in both N and N-PbCS immunized groups, excluding immune interference between N and PbCS antigens in anti-N humoral response. The anti-N antibody response was more variable among the animals than the anti-PbCS response, with even three mice from both groups that remained negative. This was not due to administration or vaccine uptake problems, as the two negative mice from the N-PbCS group (Figure 7C) had high anti-PbCS responses (Figure 7A and Figure 7B). Comparison between the anti-N and anti-PbCS antibody titers in the N-PbCS group shows a trend towards inverse correlation between these titers, the majority of mice having higher anti-PbCS than anti-N antibody titers (10 out of 14 mice, i.e. 71%; Figure 7D). This may indicate immunodominance of PbCS over measles N antigen. Lastly, all immunized mice showed anti-*P. pastoris* antibody responses towards whole (Figure 7E) or lysed *P. pastoris* (Figure 7F), and naive mice did not present anti-*P. pastoris* cross reacting antibody responses (data not shown).

Two weeks after the last immunization, all mice were challenged with 6,000 GFP^+^ *Pb* sporozoites and the parasitemia (proportion of parasite-containing RBCs) was determined at early time points during parasite exponential growth (Figure 8A). From day 3 to 6 after challenge, the parasitemia was comparable in the N, PbCS and WT groups where iRBC reduction was not statistically different from the naive control group, however the N group showed a difference from the naive group at day 5 (p<0.05; Figure 8B). In contrast, parasitemia was significantly reduced in the N-PbCS group (notably around 4–fold at day 5 post-challenge; p<0.005 at days 4 and 5, and p<0.0005 at day 6; Figure 8B). The parasitemia reduction observed in animals immunized with N-PbCS as compared to animals immunized with PbCS correlates with the induction of PbCS antibodies and confirms that multimerization of the antigen on RNPs made the difference. We observed that mice dying early (day 7–14) presented clinical signs of experimental cerebral malaria, whereas after day 20 mice died as a consequence of hyper-parasitemia (up to 35–55% of iRBCs/RBCs at day 20, and 60–70% at day 25). The parasitemia at day 5 and the day of death showed a significant inverse correlation (Spearman test; p<0.005): a low parasitemia (0–0.2%) preferentially resulted in a late death, while a higher parasitemia (>0.2%) resulted in a more rapid death associated with experimental cerebral malaria (Figure 8C). Immunization with N-PbCS yeasts increased survival since 10 out of 14 mice of this group were still alive at day 22 after challenge, while most mice from PbCS, WT and naive groups died around day 11 (Figure 8D). Nevertheless, a similar benefit on survival at later times was also observed in the group of mice immunized with yeasts expressing N only, thus indicating that N may contribute to the survival advantage observed in the N-PbCS group. Notably, in the N-PbCS group, mice with the highest anti-PbCS IgG levels had the most prolonged survival, although antibody titers were not predictive of early or late animal death outcome (Mann-Whitney nonparametric test).

## Discussion

Vaccine manufacturers largely use yeast as bioreactor for producing high amounts of low cost vaccines, the best example being the anti-hepatitis B (HBV) vaccine (ENGERIX-B®). This vaccine is based on the HBV small surface antigen (HBsAg) and is manufactured in *S. cerevisiae* yeast. Like in all yeast-based vaccines currently on the market, ENGERIX-B® HBsAg is produced and purified from yeasts. Attempts to validate *S. cerevisiae* as both an antigen bioreactor and a delivery system are currently ongoing in preclinical and clinical trials. An HCV therapeutic vaccine (GI-5005) was tested in Phase IIb, and an HBV therapeutic vaccine (GI-13000) is undergoing preclinical studies (http://www.globeimmune.com/). These vaccine candidates are based on *S. cerevisiae*, as most whole yeast-based vaccine candidates [13], [16]. Nevertheless, *P. pastoris* is an interesting alternative in the development of whole yeast vaccines, since this species introduces less post-translational modifications on heterologous antigens than *S. cerevisiae* [35]. Moreover, unlike *S. cerevisiae, P. pastoris* is particularly suitable for the fermentative growth and has the ability to reach very high cell densities during fermentation, which may improve overall protein yields [54].

With this in mind, we set up a vaccine production and delivery system based on the heterologous expression of measles virus nucleoprotein (MV-N) in *P. pastoris*. We show here that the spontaneous assembly of this protein in *P. pastoris* provides a mean to multimerize heterologous antigens. As a proof of concept, we fused to MV-N the circumsporozoite protein (CS) from *Plamodium berghei* (*Pb*), the etiologic agent of rodent malaria [46]. The CS protein is present on the surface of *Plasmodium* sporozoites (10 pg per sporozoite [55]) when they are inoculated into the skin of the host [37], [49], [56]. Antibodies [40], [41], [42] and cellular responses [42], [43], [44] elicited against CS both seem to confer protection, and the most advanced malaria vaccine candidate (RTS,S) is based on this antigen [57]. Full results from the phase III trial of RTS,S are expected in 2015. Current estimates of vaccine efficacy in the 12 months following three doses were 30–56%, depending on age group and endpoint [58]. Nevertheless, recent phase IIb analysis showed that RTS,S efficacy is inversely correlated with transmission intensity, dropping to zero in a three-year perspective [59]. This is in strong support for developing second-generation malaria vaccine strategies. In the present work, fusion of PbCS to MV-N resulted in antigen multimerization into ribonucleoprotein rod-like structures localized in the cytoplasm of recombinant yeasts. Injected subcutaneously in the absence of accessory adjuvants and at low antigen dose (130 ng PbCS per injection), the N-PbCS *P. pastoris* induced a significant delay in the emergence of parasitemia as well as prolonged survival of recipient C57Bl/6 mice following a stringent challenge consisting in the intradermal injection of a high number (6,000) of infectious *Pb* sporozoites. Comparing both the induction of specific antibodies to PbCS antigen and the outcome of experimental challenge among N, PbCS and N-PbCS immunization groups indicates that multimerization of PbCS on RNPs was necessary to significantly decrease early parasitemia and to increase mice survival. However, we also observed that measles virus N carrier protein contributes by itself, together with *Plasmodium* antigen multimerization, to parasitemia delay and to long-term mice survival. This might be due to innate immune responses or to the activation of CD8 or CD4 T-cells, although we could not analyze those responses as no T-cell responses or specific T-epitopes have been described in the C56Bl/6 mouse model of *Pb* infection [60]. Anti-PbCS IgG responses reflected unbiased contribution of Th1 and Th2 immune responses, indicating broad elicitation of the immune system in the absence of accessory adjuvants. Future studies will be dedicated to clarify the specific role in adjuvancy of N-based RNPs and of heat-inactivated whole *P. pastoris* yeast, as well as the possible role of T cell responses.

Nevertheless, this initial study shows that expression of the CS antigen in *P. pastoris* needs to be improved. It is assumed that the CS proteins from *Pb* (infecting mice) and *P. falciparum* (infecting humans) share conformational and function properties, although they present around 60% divergent amino acids sequences [39]. To determine whether CS expression in *P. pastoris* depends on specific protein sequence, we generated MV-N fusion proteins with equivalent CS domains from *P. falciparum* (strain 3D7; PfCS) and from PbCS. Full length N-PfCS (92.73 kDa) was produced in *P. pastoris* GS115 and KM71 at 97 ng/YU (data not shown), while N-PbCS (91.32 kDa) was not produced in GS115 or KM71 strains, and only at lower yield in SMD1168 strain (12 ng/YU). These data indicate that primary amino acid sequence determines the efficiency of N-CS fusion protein expression in *P. pastoris*. Yeast proteases are major actors in foreign protein degradation. As only general knowledge is available on protease targets essentially from *S. cerevisiae*, further studies are required for improving the yield of production of MV-N based fusion protein in *P. pastoris*. Nevertheless, the better production yield observed for PfCS as compared to PbCS in *P. pastoris* is favorable for developing a human vaccine relying on this strategy.

The administration regimen is an important issue for whole inactivated yeast. In this study, we show that three injections at one-week interval were necessary to elicit detectable anti-CS antibody response in most mice. In preliminary experiments we observed that three injections at two-week interval (d0, d14, d28) did not induce anti-CS antibodies or parasitemia delay (data not shown). Whether this is due to the low expression level of N-PbCS in SMD1168 strain (12 ng/YU) or to the intrinsic feature of whole recombinant *P. pastoris* needs to be addressed. Increasing antigen production yield by further selection of expressing clones is obviously required. However, frequent boosts might be needed to elicit robust long-term immune responses. Multiple doses with whole recombinant yeast were tested as administration protocols for therapeutic vaccination with myostatin [61], K-Ras oncoprotein [62] or HCV NS3 and Core (GI-5005; [15]). Notably in this last study, up to 13 weekly doses of whole recombinant yeast showed no yeast neutralization in mouse or toxicity in rabbit, and cellular immune responses increased in parallel with injection frequency [15]. However, for logistic and economic reasons, no more than three vaccine administrations can be scheduled for preventive immunization of infants living in areas of malaria endemicity. Future improvement of whole recombinant yeast such as increasing the antigen expression level, testing alternative administration pathways or yeast formulation, has to be addressed to enhance vaccine efficiency with fewer administrations.

Many immunogenic antigens from *Plasmodium* parasites were described. Although correlates of protection against malaria in humans are still elusive, the combination of *Plasmodium* antigens from both the asexual and sexual phases of infection in a single multi-stage formulation might make it possible to induce sterile immunity in humans and prevent parasite transmission. Current vaccine platforms towards malaria provide either monovalent (subunit or DNA/viral vector vaccine candidates), or multivalent formulations (whole organism vaccine candidates: i.e. genetically attenuated parasites, radiation-attenuated sporozoites, and live-parasite immunization under antimalarial therapy) [63]. The monovalent vaccine platforms, even in heterologous immunization protocols, result in poorly diversified antigen combinations and show at best partial protection towards infection. By providing all antigens from *Plasmodium* at specific replication stages, same vaccine protocols from the multivalent vaccine strategies are successful in eliciting sterile immunity. However, whole organism vaccine candidates are still limited by hardly solvable logistic problems for large-scale production, distribution and delivery in developing countries.

Mixtures of whole recombinant yeast clones, each expressing relevant *Plasmodium* antigens for the asexual and sexual stages, provide the means to deliver multi-antigen cocktails in a vaccine formulation which can guarantee low costs of production and be independent from cold chain constraints for vaccine delivery in developing countries.

## Materials and Methods

### Recombinant Yeast Production and Characterization

The N and the PbCS nucleotide sequences were synthesized and optimized for expression in *P. pastoris* (GeneScript), and cloned within *EcoRI* and *NotI* restriction sites into the pPIC3.5K plasmid (Invitrogen) for yeast expression under the control of the methanol-inducible AOX1 promoter. In the N-PbCS fusion protein, a linker of 7 amino acids (GAAGAGA) was inserted between the N and PbCS genes. GS115, KM71 and SMD1168 yeast strains were transformed by electroporation and plated on RDB plates (histidine-deficient medium) for the first round of clone selection (*HIS^+^* clones). Screening of clones with multiple inserts was performed on YPD-Geneticin plates at a final antibiotic concentration of 0.25 to 4.0 mg/ml (G8168-100, Sigma-Aldrich). Details on yeast culture mediums and plates are given in Invitrogen User Manuals for *P. pastoris*.

Kinetics and levels of N, PbCS and N-PbCS protein expression were monitored upon methanol induction. Yeast clones were cultured in BMG medium over weekend, then transferred to the BMM medium and protein production was induced and maintained by adding 0.5% methanol to cultures every 24 h. Before lysis, yeast cells were quantified by spectrophotometer analysis at OD_600 nm_. Collected culture samples were lysed every 24 h using acid-washed glass beads (425–600 µm; G8772 Sigma-Aldrich) and Breaking Buffer (Invitrogen). Following mechanic lysis, yeast extracts were centrifuged at 134 *g* for 10 minutes, and then supernatants clarified by centrifugation at 371 *g* for 15 minutes. Western blot (WB) was performed in denaturing conditions on 4–12% Bis-Tris polyacrylamide gels with XT MOPS buffer (Criterion 345-0123, Bio-Rad) using the Color Plus Prestained Protein Ladder (7–200 kDa; P7711 BioLabs), nitrocellulose membranes (HybondTM-C Extra RPN303E; Amersham Biosciences), and as primary antibodies the anti-N clones 25 or 120 (a gift from Mathias Faure and Chantal Rabourdin-Combe; [64]) or the anti-PbCS antibody obtained through the MR4 as part of the BEI Resources Repository, NIAID, NIH: *Mus musculus* (B cell); *Mus musculus* (myeloma) 3D11, MRA-100, deposited by V Nussenzweig. Primary antibodies were at 1/1,000 dilution over night at 4°C and the secondary HRP-conjugated sheep anti-mouse IgG antibody (GE Healthcare UK Limited, NA931V) at 1/5,000 dilution for 1h30 at room temperature. In quantitative WB, selected clones were induced in BMM and cultures stopped at 54 h. Yeast was quantified (yeast unit; YU) by spectophotometer analysis (OD_600 nm_) and lysed. Lysed samples were diluted as indicated, and loaded on gel in parallel to the N standard protein (GenScript) at predefined quantities (10 to 20 ng). The anti-N clone 25 was used as primary antibody. N and N-PbCS band intensities were quantified by the Luminescent Image Analyzer LAS-1000 plus (FUJIFILM) and reported on the N standard curve. Total soluble proteins (TSP) in lysates were measured by Bio-Rad Bradford Assay.

### Size of MV RNPs Expressed in *Pichia pastoris*

After methanol induction for 54 h, yeast cultures were stopped on ice and samples (4,325 YU) were lysed and resuspended in 2 ml suspension buffer (SB: TrisHCl 25 mM pH 7.5, NaCl 50 mM, EDTA 2 mM in UltraPure™ DNase/RNase-Free Distilled Water) supplemented with anti-protease cocktail (Roche) and rRNasin RNase Inhibitor (Promega). The 2 ml samples were loaded on 9 ml 20% sucrose cushion in SB and centrifuged in SW41 Ti rotor for 1 h at 36,000 rpm at 4°C. Fractions of 1 ml were collected using the Masterflex® L/S® compact pump sampling machine (Cole-Parmer). Pellets were resuspended in 1 ml SB. Each aliquot including the pellet was analyzed for total soluble proteins (TSP), total RNA and N or N-PbCS protein concentrations by Bio-Rad Bradford Assay, NanoDrop 1000 Spectrophotometer, and anti-N or anti-PbCS sandwich ELISA. PCR analysis on lysed yeast cultures before and after centrifugation at 134 g and on clarified lysates was performed by a classical protocol using Taq DNA polymerase from Invitrogen and the 5′ AOX1 (Invitrogen) and the 3′ NOPT-INTER (5′- TTGTTCAGTCTGACCAGTCTC) primers resulting in a 437-nucleotide band on recombinant yeast genome. Anti-N and anti-PbCS sandwich ELISA were performed by coating in sodium carbonate buffer (pH 9.6) 0.5 µg/ml of the mouse anti-N (MAB8906 Millipore) or 1/2,000 dilution of the MR4-100 anti-PbCS monoclonal antibody, and using the anti-N rabbit polyclonal IgG antibody (ABIN346975 Antibodies-Online GMBH) at 1/10,000 in 1xPBS as primary antibody, and the anti-rabbit IgG-HRP (NA934V Amersham Biosciences) at 1/7,000 dilution in 1xPBS as secondary antibody. Anti-N ELISA positive controls were SMD1168 expressing N protein, lysed and diluted at 1/200, 1/400 and 1/800 in 1xPBS. Anti-PbCS ELISA positive control was N-PbCS SMD1168 lysate (1/200) before loading on ultracentrifugation tube. ELISA plates were read with the EnSpire 2300 Multilabel Reader (Perkin Elmer) at OD_450 nm_, using OD_620 nm_ as reference wavelength. Fractions and pellets collected from ultracentrifugation tubes with no yeast (SB only loaded on 20% sucrose) showed negative background of reagents in all the performed tests (ELISA, NanoDrop and Bradford).

A sedimentation calculation routine (developed by Dr Raynal, Institut Pasteur, and provided on demand) was set up in Microsoft Excel using classical Svedberg equations to predict the distance (cm) at which the protein of interest migrates from the upper surface of solutions in ultracentrifugation tubes. The calculation takes into account: (i) the protein mass and structure to estimate the vbar and Sw20 sedimentation parameters; (ii) ultracentrifugation tube characteristics and rotor diameter; (iii) sucrose percentage and volume for each solution phase; (iv) ultracentrifugation time; and (vi) rotation speed (rpm).

### Electron Microscopy

SMD1168 yeasts expressing N or N-PbCS were lysed and clarified as described above. EM was directly performed on N clarified lysate, while the N-PbCS clarified lysate was concentrated by ultracentrifugation on 20–60% sucrose gradient for 1 h30 at 32,000 rpm (SW32 Ti). The fractions at the interphase were collected, and further ultracentrifuged on 30% sucrose cushion for 4 h at 32,000 rpm (SW32 Ti) to collect the pellet. Samples were spotted on glow discharged carbon coated grids (EMS, USA) and negatively stained with NanoW (Nanoprobes, USA) [65]. Samples were then observed at 120 kV with a Philips/FEI CM 12 transmission electron microscope. Images were recorded using a KeenView camera (OSIS, Germany) and ITEM software (OSIS, Germany). RNP length and diameter were estimated as the average measures of 50 particles counted manually. Measurement standard deviation was 5%.

### Immunofluorescence Analysis

After induction of protein expression, 50 YU per sample were fixed by 3.7% formaldehyde. Cell wall was digested by zymolyase (Sigma-Aldrich: L2524-50KU), and cells were fixed again by methanol/acetone and attached to microscope slides as described [66]. Cells were labeled with a rabbit polyclonal anti-MV-N (Covalab pab0035; 1/500 dilution) or MRA-100 mouse anti-PbCS monoclonal antibodies (1/1,000 dilution), then Alexa 488 goat anti-rabbit IgG (H+L) (Invitrogen A-11008; 1/500 dilution) or CY3-AffinityPure F(ab’)2 Frag goat anti-mouse IgG (Jackson ImmunoReasearch 115-166-072; 1/1,000 dilution) as secondary antibodies. Bright field and fluorescence images were acquired on a motorized inverted wide field fluorescence microscope. The system was controlled by the AxioVision software (Release 4.8.2.0, Zeiss) and was composed of a motorized inverted microscope (AxioObserver Z.1, Zeiss) equipped with a halogen illuminator (HAL100, Zeiss), a metal halide illuminator (HXP120, Zeiss) and a CCD camera (AxioCam MR, Zeiss). DAPI, Alexa Fluor and Cy3 were detected with specific filter sets. Stacks of 6 focal planes spaced 0.5 µm apart were acquired with a 100x oil objective (EC Plan-Neofluar 100x/1,30 Oil Iris, Zeiss). Images were then processed with the ImageJ software [67].

### Heat-inactivation of *Pichia pastoris*

GS115, KM71 or SMD1168 yeast strains were cultured in YPD medium at 30°C and 250 YU were pelleted at 134 *g* for 10 minutes, medium was carefully removed and the yeast pellet treated at indicated temperatures and time points in a water bath. Heat-inactivation was stopped by transferring samples on ice. One YU of each sample was then plated on YPD/agar and cultured for 7 days at 30°C. The viability test was performed by adding 20 µl of methylene blue solution (Sigma-Aldrich; 0.05 mmol^−1^ in PBS pH 7.2) to the same volume of yeast cell suspensions and dead cell counting was performed under optical microscopy. For immunizations, whole SMD1168 wild type yeast or yeast expressing N, PbCS or N-PbCS (54 h cultures) were heat-inactivated at 60°C for 45 minutes and resuspended in 1xPBS at 30 YU/50 µl.

### Mice Immunization, Flow Cytometry, Survival Rate and ELISA Analyses

Six weeks old C56Bl/6 females were housed and included in experimental protocol groups following the European Directive N° 2010/63/UE. The experimental protocol was submitted and approved by the Ethic Comity Ile-de-France – Paris 1 (N° 2012-0009). All the experimenters had a regulatory authorization for animal handling delivered by the accredited French authorities and accepted by Institut Pasteur Animal Facility. All efforts were made to minimize animal suffering and to reduce the number of animals used. Mice were injected subcutaneously (50 µl) in correspondence of inguinal lymph nodes and bleedings (100 µl) were performed 3 days before the first immunization (day minus 3) or 6 h before next immunizations. Following the last bleeding (d42), mice were challenged at d43 with 6,000 sporozoites per mouse (1 µl, injected intradermally in the posterior footpad). Sporozoites were freshly collected by salivary gland dissection from *Anopheles stephensi* infected with *Pb* ANKA strain expressing the GFP (GFP^+^) under the control of the *hsp70* promoter [52]. After challenge, mice were monitored daily during two weeks for clinical signs of neurological disorder, then twice a day up to day 29 for the animals that recovered from initial symptoms. To determine the clinical benefits of vaccination and avoid arbitrary outcomes, mice were not euthanized before day 15. Beyond day 15, surviving mice did not show any predictive symptom till sudden death, despite hyper-parasitemia. In the present study, most of the mice that survived beyond day 15 did recover from clinical symptoms and in these mice we did observe early signs of cerebral malaria. Signs of cerebral malaria were motor troubles, ruffled fur and sometimes convulsions. If mice showing these signs had been euthanized before day 15, or had received any treatment, the outcome of the experiment (possibility to recover from cerebral malaria) would have been arbitrarily changed. No significant weight change was observed. Parasitemia level was not correlated to the day of death. Identified moribund mice were euthanized by C0_2_ treatment in an appropriate chamber.

Blood samples (2 µl) were collected from day 3 to 7, diluted in 600 µl 1xPBS and analyzed in plates by Fluorescence Activated Cell Sorting (FACS; MacsQuant, Miltenyi Biotec). Doublets and clusters of red blood cells (RBCs) were excluded from counts. Single GFP^+^ RBCs (infected RBC, iRBCs) among total RBCs were estimated and data analyzed by the MACSQuantify™ Software.

For IgG quantification in blood, sera from bleedings were separated from blood samples by Capiject T-MG Capillary Blood collection System (Terumo Medical Corporation) and stored at −20°C untill ELISA tests. The anti-PbCS IgG ELISA was performed by coating wells with 50 µg of recombinant PbCS protein produced at the Recombinant Protein and Antibodies Production Core Facility of the Institut Pasteur by J. Bellalou and V. Bondet, using the BioPod F800 microfermentor battery (Fogale Nanotech) [68]. The anti-PbCS monoclonal antibody from the MR4-100 reagent was used for positive controls at 1/4,000 and 1/10,000 dilutions. Anti-N IgG ELISA was performed by coating in wells 50 µg of >70% pure recombinant N protein (Genscript) and using the monoclonal mouse anti-N primary antibody (MAB8906, Millipore) at 1/5,000 and 1/20,000 dilutions in 1xPBS, Tween 0.05% and BSA 0.5% for positive controls. Anti-*P. pastoris* IgG ELISA was performed by coating 25 YU of whole or lysed wild type *P. pastoris* per well in sodium carbonate buffer (pH 9.6). Yeast was previously cultured, inactivated and lysed as described. Saturation of wells by whole or lysed yeast was determined by using an anti-*P. pastoris* rabbit polyclonal antibody (BP2240, Acris Antibodies) at 1/200 dilution and an anti-rabbit IgG-HRP (NA934V Amersham Biosciences) at 1/10,000 dilution. Serial dilutions of serum samples (1/100, 1/1,000, 1/10,000 and 1/100,000 for anti-PbCS and anti-N; 1/300 and 1/1,000 for anti-*P.* pastoris) were incubated on plates then HRP-conjugated sheep anti-mouse IgG secondary antibody (NA931V, GE Healthcare UK Limited) was used at 1/5,000 dilution in PBS together with 3,3′,5,5′-Tetramethylbenzidine substrate (TMB; Sigma-Aldrich) for detection. ELISA development was stopped after 5 minutes with 2N H_2_SO_4_ and plates were read at OD_450 nm_, using OD_620 nm_ as reference wavelength. In ELISA determination of IgG isotypes, the polyclonal goat anti-mouse ads-HRP IgG (1030-05: dilution 1/8,000), IgG1 (1070-05; dilution 1/4,000), IgG2a (1080-05; dilution 1/4,000) and IgG2b (1090-05; dilution 1/4,000) from Southern Biotech were used as secondary antibodies. Sera were diluted by two folds from 1/50 to a maximum of 1/614,400. Titers were determined as the inverse of the highest sample dilution for which the OD_450 nm_ signal was greater than the cut off (the mean optical density plus 3 times the standard deviation of pre-immune control sera from mice under study). Mann-Whitney nonparametric and Spearman tests were performed using GraphPad Prism version 5.0 b for Mac OS X (GraphPad Software, San Diego California USA, www.graphpad.com), and the Wilcoxon Two Sample test (http://www.fon.hum.uva.nl/Service/Statistics/Wilcoxon_Test.html).

## Supporting Information

Figure S1 **Optimized nucleotide sequence of the N (A) and PbCS (B) proteins for expression in GS115, KM71 and SMD1168** ***P. pastoris*** **strains.** The nucleotide sequence of the linker between N and PbCS is given in the box (**B**); (**C**) Kyte-Doolitle hydropathy profile (DNA Strider1.4f18) of N-PbCS: negative values correspond to hydrophilic amino acid motifs.
(TIF)

Figure S2 **Total RNA (A, B and C) and Total Soluble Protein (TSP; D, E and F) in fractions (Fr) and pellets from ultracentrifuged samples (U) in duplicate.** (**A** and **D**) SMD1168 *P. pastoris* transformed with pPIC3.5K without insert; (**B** and **E**) SMD1168 *P. pastoris* expressing N; (**C** and **F**) SMD1168 *P. pastoris* expressing N-PbCS. SB: suspension buffer. PCR analysis targeting the gene insert demonstrated the absence of genomic DNA (of nucleus origin) in samples analyzed by NanoDrop for total RNA content.
(TIF)

Figure S3 **Heat-inactivation of** ***P. pastoris*** **GS115, KM71 and SMD1168.** The hyphen (-) corresponds to incomplete inactivation and “n.v.” to not-viable yeast. In the figure: test of *P. pastoris* reproductive activity on YPD plates following heat-inactivation. Each spot corresponds to 1 YU (5×10^7^ cells), out of 250 YU samples, loaded on a YPD/agar plate and cultured over 7 days at 30°C. GS115 heat-treated samples are numbered on horizontal lines from 1 to 8, KM71 from 9 to 16 and SMD1168 from 17 to 24. (**A**) GS115, (**B**) KM71, and (**C**) (SMD1168) samples were not submitted to heat-treatment, while all the other spots were submitted to 58°C for 45 minutes (1, 2, 9, 10, 17, 18) or 60 minutes (3, 4, 11, 12, 19, 20), and to 60°C for 45 minutes (5, 6, 13, 14, 21, 22) or 60 minutes (7, 8, 15, 16, 23, 24). Untreated samples actively grew (A, B and C), while all heat-inactivated samples (from 1 to 24) were completely arrested in their reproductive activity (visible spots correspond to 1 YU loaded on plates).
(TIF)
