# Supplementary figures and images for: Whole Pichia pastoris Yeast Expressing Measles Virus Nucleoprotein as a Production and Delivery System to Multimerize Plasmodium Antigens
Source: PLoS One. 2014 Jan 27;9(1):e86658. doi: 10.1371/journal.pone.0086658 (PMC3903550; doi:10.1371/journal.pone.0086658)

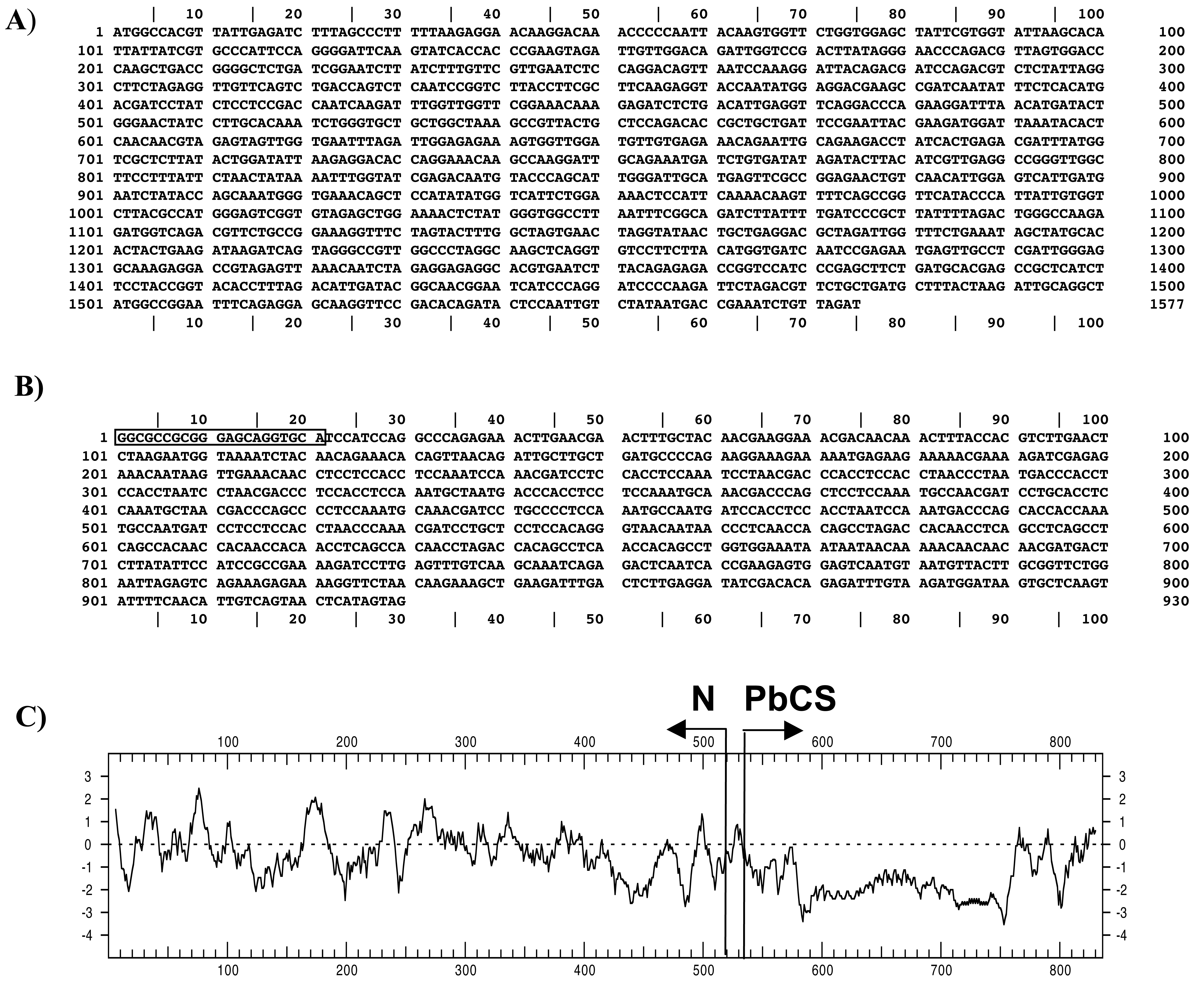

Supplement: Figure S1 — Optimized nucleotide sequence of the N (A) and PbCS (B) proteins for expression in GS115, KM71 and SMD1168 P. pastoris strains. The nucleotide sequence of the linker between N and PbCS is given in the box (B); (C) Kyte-Doolitle hydropathy profile (DNA Strider1.4f18) of N-PbCS: negative values correspond to hydrophilic amino acid motifs. (TIF) [file pone.0086658.s001.tif]

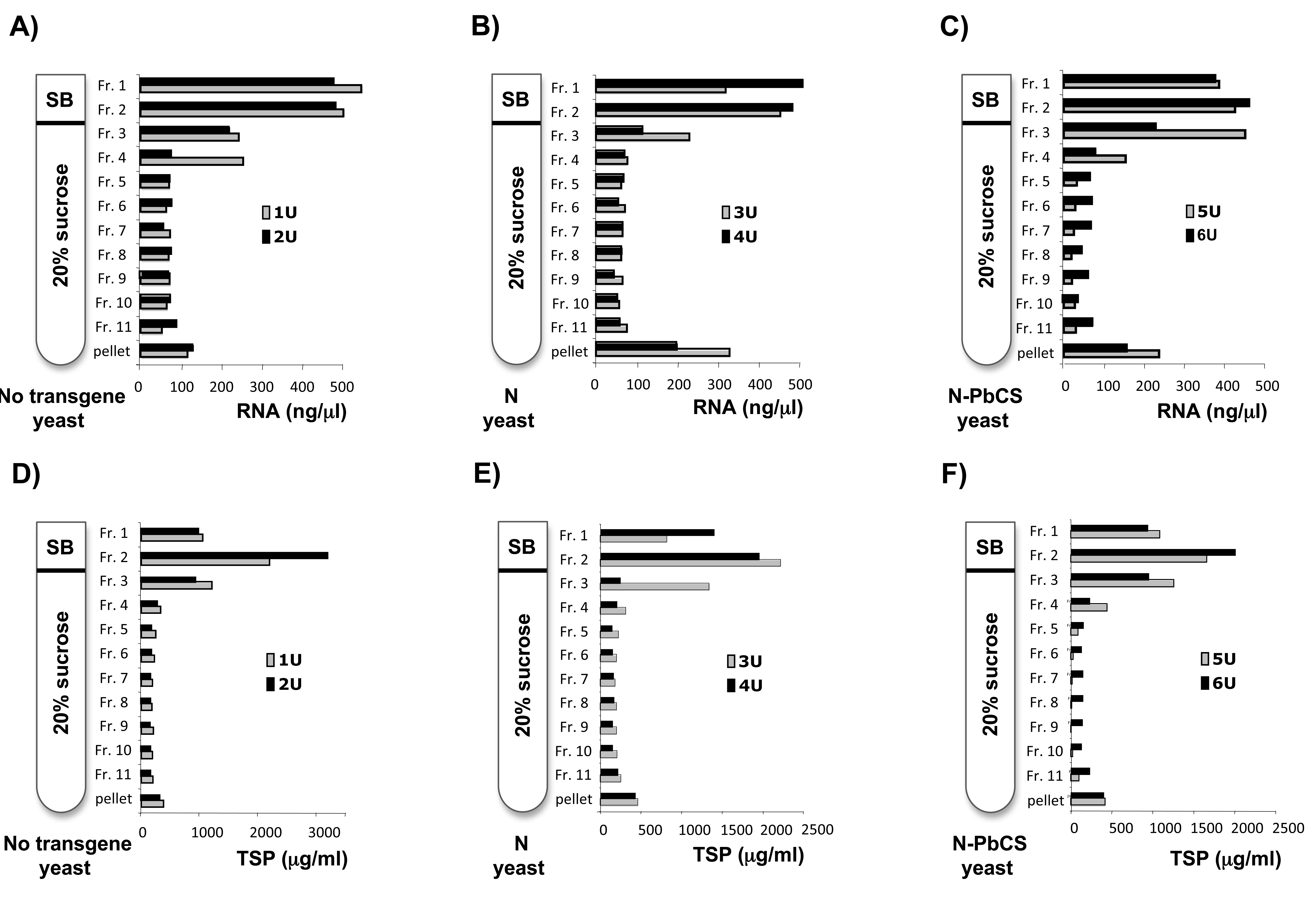

Supplement: Figure S2 — Total RNA (A, B and C) and Total Soluble Protein (TSP; D, E and F) in fractions (Fr) and pellets from ultracentrifuged samples (U) in duplicate. (A and D) SMD1168 P. pastoris transformed with pPIC3.5K without insert; (B and E) SMD1168 P. pastoris expressing N; (C and F) SMD1168 P. pastoris expressing N-PbCS. SB: suspension buffer. PCR analysis targeting the gene insert demonstrated the absence of genomic DNA (of nucleus origin) in samples analyzed by NanoDrop for total RNA content. (TIF) [file pone.0086658.s002.tif]

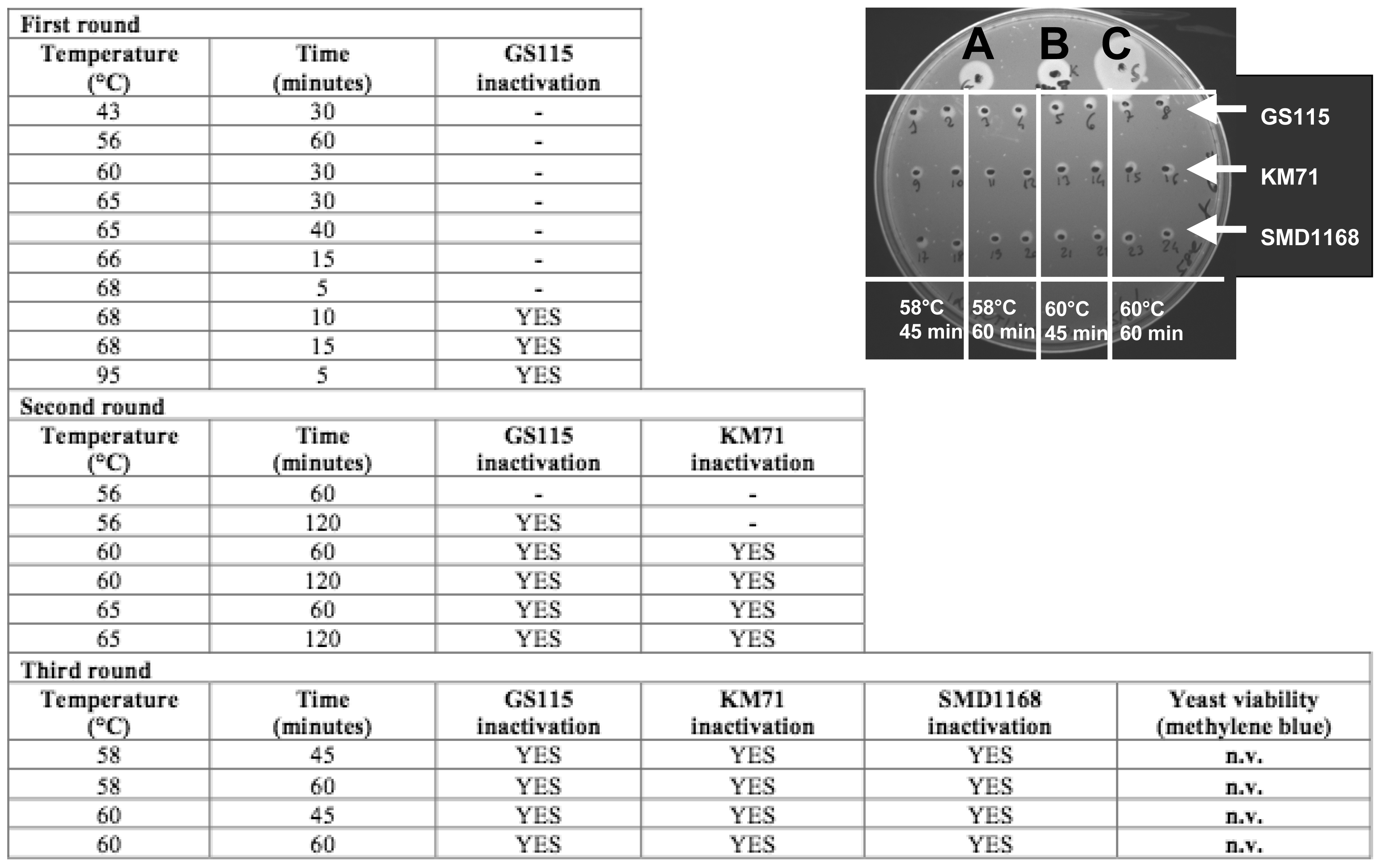

Supplement: Figure S3 — Heat-inactivation of P. pastoris GS115, KM71 and SMD1168. The hyphen (-) corresponds to incomplete inactivation and “n.v.” to not-viable yeast. In the figure: test of P. pastoris reproductive activity on YPD plates following heat-inactivation. Each spot corresponds to 1 YU (5×10^7^ cells), out of 250 YU samples, loaded on a YPD/agar plate and cultured over 7 days at 30°C. GS115 heat-treated samples are numbered on horizontal lines from 1 to 8, KM71 from 9 to 16 and SMD1168 from 17 to 24. (A) GS115, (B) KM71, and (C) (SMD1168) samples were not submitted to heat-treatment, while all the other spots were submitted to 58°C for 45 minutes (1, 2, 9, 10, 17, 18) or 60 minutes (3, 4, 11, 12, 19, 20), and to 60°C for 45 minutes (5, 6, 13, 14, 21, 22) or 60 minutes (7, 8, 15, 16, 23, 24). Untreated samples actively grew (A, B and C), while all heat-inactivated samples (from 1 to 24) were completely arrested in their reproductive activity (visible spots correspond to 1 YU loaded on plates). (TIF) [file pone.0086658.s003.tif]
